# Unlocking Holocentric Chromosomes: New Perspectives from Comparative and Functional Genomics?

**DOI:** 10.2174/138920212801619250

**Published:** 2012-08

**Authors:** Mauro Mandrioli, Gian Carlo Manicardi

**Affiliations:** 1 Dipartimento di Biologia, Università di Modena e Reggio Emilia, Via Campi 213/D, Modena, Italy; 2 Dipartimento di Scienze Agrarie e degli Alimenti, Università di Modena e Reggio Emilia, Reggio Emilia, Italy

**Keywords:** Holocentrism, holokinetic chromosomes, genome size, gene distribution, GC content, kinetochores, condensin, chromosome rearrangements.

## Abstract

The presence of chromosomes with diffuse centromeres (holocentric chromosomes) has been reported in several taxa since more than fifty years, but a full understanding of their origin is still lacking. Comparative and functional genomics are nowadays furnishing new data to better understand holocentric chromosome evolution thus opening new perspectives to analyse karyotype rearrangements in species with holocentric chromosomes in particular evidencing unusual common features, such as the uniform GC content and gene distribution along chromosomes.

## THE HOLOCENTRIC-HOLOKINETIC CHROMOSOMES

The centromere of eukaryotes was identified as the primary constriction of chromosome visible by light microscopy [1]. Successive molecular and cytogenetic analyses characterized centromeres as heterochromatic chromosomal domains that direct the formation of kinetochore, representing the primary chromosomal attachment site for spindle microtubules [2].

**Fig. (1):**
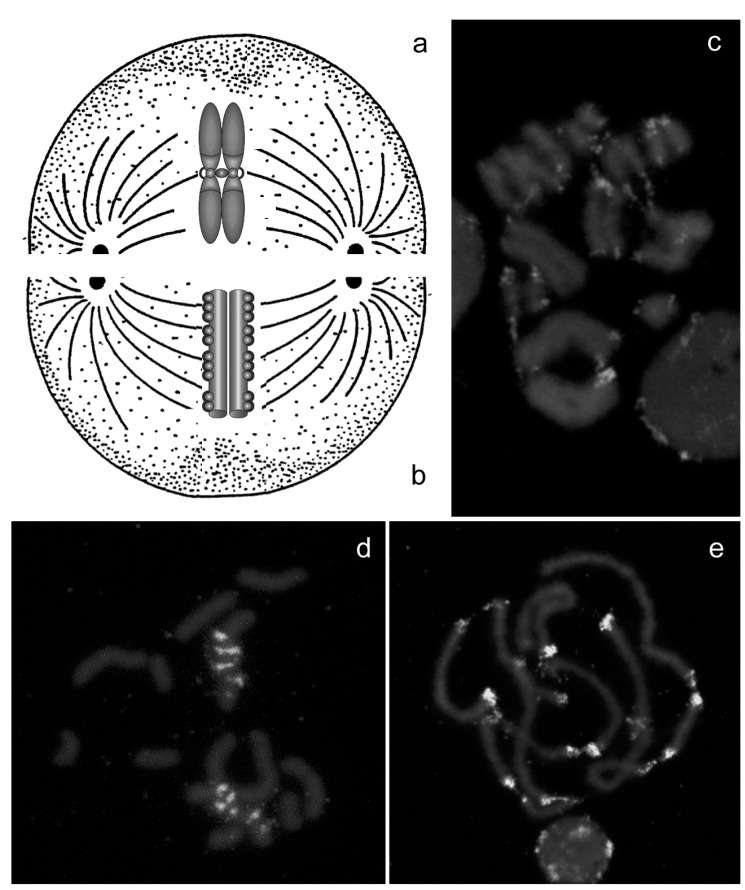
Monocentric (**a**) and holocentric chromosomes (**b**) differ for the presence of a localized centromere. In view of diffuse kinetic activity, holocentric chromatids move apart in parallel and do not form the classical V-shaped figures typical of monocentric (**c**) and do not have a preferential localization of heterochromatin that can be present in different chromosomal areas, as showed by *in situ* hybridization with the *Hind*200 satellite DNA (**d**) and the subtelomeric repeat (**e**) probes on the holocentric chromosome of the aphid *Myzus persicae*.

Despite the functional importance of centromeres, cytologists have long observed that a primary constriction cannot be observed on chromosomes of all the species and that, in some taxa, spindle microtubules can attach along the entire chromosome length [3]. Starting from the beginning of ’60, it has been therefore necessary to distinguish monocentric chromosomes that attach to microtubules at single region (the centromere) and move toward the pole during anaphase with the centromere leading (Fig. **1a**), in contrast to holocentric chromosomes whose kinetochores are diffuse so that they bind to microtubules along their entire length and move broadside to the pole from the metaphase plate (Figs. **1b-e**) [3-5]. These chromosomes are also termed holokinetic, because chromatids move apart in parallel and do not form the classical V-shaped figures typical of monocentric chromosomes [5].

Ultra-structural studies confirmed the holocentric-holokinetic architecture of the chromosomes in some species, as reported in the mite *Tetranychus urticae* whose chromosomes have points of attachment to the spindle microtubules extending across the entire chromosomal length [3, 6]. The holokinetic nature has also been confirmed by the evidence that experimentally induced chromosome fragments continue to attach to the spindle and segregate correctly [3, 4].

Chromosomes with diffused centromeric activity have been found in protista, plants and animals so that this particular type of organization has been described in almost all the eukaryotic taxa so far examined, with the exception of echinoderms and cordata [2, 5, 7-15]. In particular, holocentric chromosomes have been observed in different metazoa, including insects (such as moths and butterflies, aphids, coccids, earwigs and triatomines), nematodes and arachnids [7-15].

In an evolutionary context, it has been observed that the holocentric condition is present in each principal knot of the eukaryotic phylogenetic tree, but the discontinuous distribution of monocentrism and holocentrism in protista, animals and plants put in discussion the problem of their origin and evolution. Indeed their scattered presence could be the result of convergent evolution so that holocentric chromosome probably arose multiple times during evolution [2, 5, 16]. However, at present we cannot exclude that the ancestral eukaryotic chromosomes may have been holocentric, in which case the restriction of kinetic activity to a specialized region must have been a frequent evolutionary event [2]. Interestingly, the existence of related plant species with either holocentric or monocentric chromosome architectures offered an unique possibility for investigating the evolution of holocentrism [17], but up till now a clear scenario cannot be detailed in order to explain the origin of these distinct chromosome architectures in the eukaryotic kingdom.

According to literature data, one potential advantage associated with the holocentric chromosome architecture is related to double-strand DNA breaks. Indeed double-stranded breaks in monocentric chromosomes bring to chromosomal fragments that cannot be properly inherited by cells due to their inability to attach to spindle microtubules. In contrast, fragments of holocentric chromosomes, could properly segregate and be stably maintained since they have microtubule attachments sites along their entire length. However, the wide prevalence of species with monocentric chromosomes suggests that advantages gained with the holocentric chromosome architecture are counterbalanced by some disadvantages, probably related to difficulties in the segregation of recombined holocentric chromosomes during meiosis [2].

The ability of stabilizing double-strand DNA breaks observed in holocentric chromosomes might have arisen as a defense mechanism against the production of chemicals able to induce DNA damages by several plants. Nicotine, for instance, is a naturally occurring alkaloid found primarily in members of the solanaceous plant family (including *Nicotiana tabacum*) that cause replication fork stress bringing to different DNA damages, including chromosomal fragmentations [18, 19]. Similar effects have been also reported by other plant-produced molecules, such as caffeine and ethanol [18, 19]. In view of these effects, the presence of holocentric chromosome in phytophagous insects, such as aphids and lepidopteran species, could be a response to the clastogenic effects of some molecules produced by the plant tissue during the insect feeding [20].

A different hypothesis has been suggested in order to explain the occurrence of holocentric chromosomes in nematodes. In particular, as suggested by Pimpinelli and Goday [16], a single inappropriate cell death can have severe consequences in nematode development that is typically characterized by fixed lineages. Holocentrism in nematodes could therefore avoid the disastrous consequences of unrepaired chromosome breakage events during nematode development [16].

Surprisingly, despite their diffuse presence in protista, plants and animals, holocentric chromosomes have been regarded with a mixture of curiosity and suspicion [2], so that studies concerning chromosome structure have been mainly concentrated upon eukaryotes having monocentric chromosomes, whereas species possessing holocentric /holokinetic chromosomes have been almost neglected. At present, the availability of different wholly sequenced genome projects (such as those of *Caenorhabditis elegans*, *Tetranychus urticae*, *Acyrthosiphum pisum* and *Bombyx mori*), together with other ongoing projects (including those of the tick *Ixodes scapularis* *and Varroa destructor*), put comparative genomics in a pivotal position to deeply investigate the origin and evolution of holocentric chromosomes with particular regard for Metazoa.

## HOLOCENTRISM AND GENOME SIZE

Eukaryotic genome size data are becoming increasingly important for comparative research and, considering that eukaryotic genome size databases represent some of the broadest available genetic datasets, it may be interesting to better understand the phenotypic effect and consequences of genome size variation in eukaryotes. Even if several associations need to be further studied, diverse papers identified some characteristics that co-vary with genome size [21, 22]. For example, a positive relationship between body size and genome size has been reported in aphids [23] and mosquitoes [21] and, similarly, cell size appears to be positively correlated with genome size in a variety of taxonomic groups [21]. Moreover, flying insects have extremely high mass-corrected metabolic rates and generally they have small genomes [21]. Lastly, Gregory [21] proposed that holometabolous groups (characterized by complete metamorphosis) have smaller genomes than those that are hemimetabolous or ametabolous. Specifically, the holometabolous orders possess genomes smaller than 1C = 2 pg whereas the ametabolous and hemimetabolous taxa mainly possess genomes that range from 1 pg to 17 pg. 

The adaptive significance of these associations, if any, and their general evolutionary significance in different taxa remains at present unclear, but eukaryotic genome size data could represent a good starting point also to face the evolution of complex traits, including for instance eusociality or peculiar genome/chromosome architecture [24].

Recently, Grbic *et al.* [25] reported that multiple characteristics of the spider mite *Tetranychus urticae* genome (including its compact size of 90Mb) correlate with the holocentric nature of the chromosomes. This is an intriguing suggestion that was, for instance, supported by the presence of holocentric nature also in the small genome of the nematode *Caenorhabditis elegans* (97Mb) [22]. Is there any constraint that limits the genome size of organisms possessing holocentric chromosomes? 

As previously reported, holocentric chromosomes have been observed in different taxa. Lepidoptera, according to Gregory and Hebert [26], have a quite variable genome size since it ranges from 0.29 pg in the monarch butterfly* Danaus plexippus* to 1.94 pg in the least-marked euchlaena* Euchlaena irraria*. A smaller range has been reported in aphids (Hemiptera: Aphididae), whose genome size ranges from 0.18 pg of *Eoessigia longicauda* to 0.89 pg reported in *Gypsoaphis oestlundi* [23] and triatomines (Hemiptera: Reduviidae), whose genome size varies from 0.92 pg in *Triatoma dimidiata* to 2.90 pg measured in *T. delpontei* [27, 28].

Holocentric chromosomes has been also observed in some arachnid species belonging to families Segestridae, Buthidae, Dysderidae and Pholcidae, whose genome size ranges from 0.08 pg reported in the two-spotted spider mite *Tetranychus urticae* to 3.41 pg in *Amblyomma americanum* [29], and in nematodes that present a genome size ranging from 0.02 pg in the plant-parasitic nematode *Pratylenchus coffeae* to 2.50 pg in the horse roundworm *Parascaris univalens* [30].

The analysis of the genome size variation across a wide range of taxonomic levels clearly revealed that the presence of a small genome is not typical of organisms having holocentric chromosomes neither any bias is present correlating small genome size and holocentrism. A different scenario is therefore emerging rejecting the idea of a selection against large genomes in organisms possessing holocentric chromosome.

## HOLOCENTRISM, GC CONTENT AND GENE DISTRIBUTION ON CHROMOSOMES

**Fig. (2):**
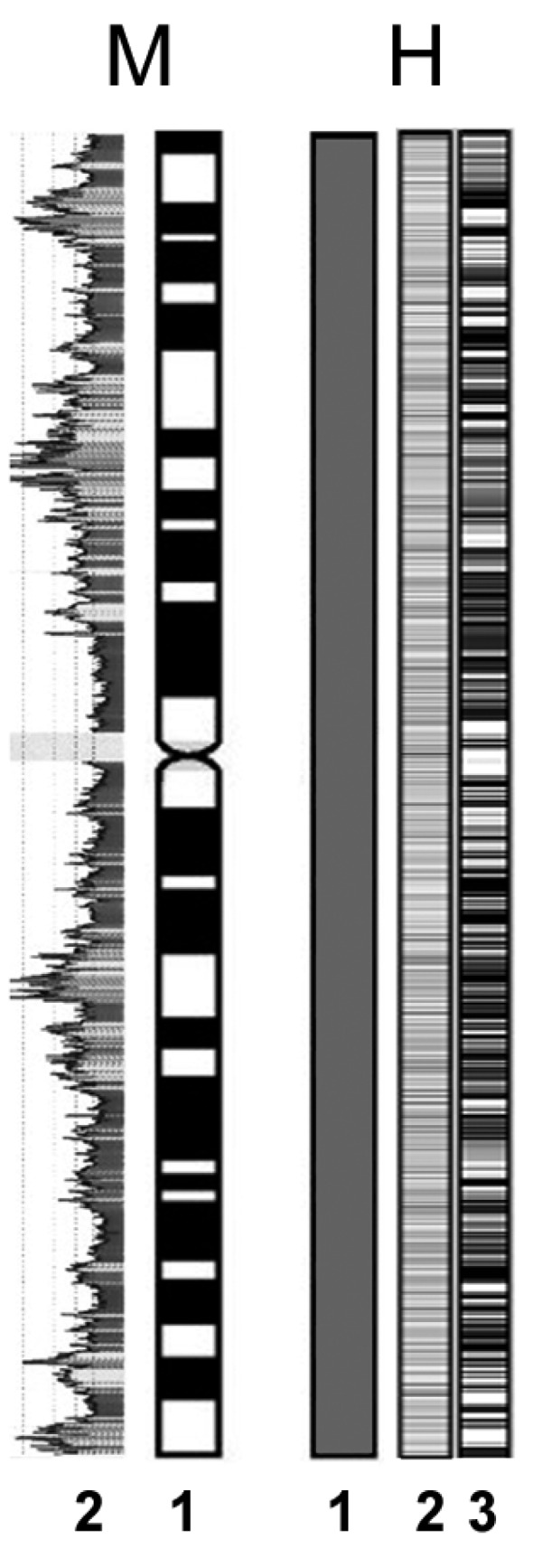
In monocentric chromosomes (**M**) an evident compartimentalization of chromosome has been observed (**1**) and a strong relationship between banding and GC% content (**2**) has been observed. In holocentric chromosomes (**H**) classical banding techniques generally fail in evidencing bands (**1**) as a consequence of a uniform distribution of genes (**2**) and GC% content (**3**).

It is generally assumed that eukaryotic chromatin possesses some degree of compartimentalization [31], so that, even if the distribution of genes on monocentric chromosomes may vary significantly among species, it is generally non-uniform. In human chromosomes [31], for instance, gene density is very high in GC-rich isochores and very low in GC-poor isochores (Fig. **2**). Furthermore, generally each monocentric chromosome possesses a large tract of gene-poor centromeric and peri-centromeric heterochromatin.

The analysis of the distribution of the GC content in the holocentric chromosomes of the spider mite *T. urticae* and the nematode *C. elegans* revealed that in both species it is essentially unchanged across all the chromosomes, unlike in vertebrate and yeast genomes [32]. Interestingly, even if the GC content may be significantly different in species with holocentric chromosomes (ranging from 29.6 in the aphid *A. pisum* to 36% in *C. elegans*), its amount is fairly constant on chromosomes [25, 33] (Fig. **2**).

Gene density is also fairly constant across chromosomes, although some differences are apparent in *C. elegans* between autosomes and the X chromosome, where genes are at a lower density and more evenly distributed [25, 33].

So far there are no detailed information regarding the distribution of genes on arthropod holocentric chromosomes, but previous cytogenetic results suggested that in the aphid *Megoura viciae* the distribution of genes was uniform throughout all autosomes, with some differences related to X chromosomes where a certain degree of compartimentalization has been observed [34].

The peculiar distribution of the GC content and genes is not typical of all the small sized genomes since, for instance the human body louse *Pediculus humanus* (whose genome size is 100Mb) has 95% of the genes are concentrated in an half of the genome [35]. A non-uniform distribution is also present in *Drosophila melanogaster* and *Anopheles gambiae, *where three isochore families have been identified with gene density increasing in the GC-rich isochores [36].

In view of the common and diffuse presence of a non-uniform gene distribution in Metazoa, it emerges that the uniform gene distribution is peculiar of species with holocentric chromosomes and that chromosome rearrangements, facilitated by the holocentric nature of chromosomes, disrupt the gene-rich chromosomal regions bringing to a uniform gene distribution.

A further shared element in species having holocentric chromosomes is that highly repetitive sequences, that are generally characteristic of centromeres in other organisms, are arranged frequently in many tandem repeats found scattered among chromosome Figs. (**1d-e**). According to data discussing the distribution of heterochromatin in taxa possessing holocentric chromosomes, heterochromatin has generally a telomeric and, sometimes, intercalary localization on chromosome, but several chromosomes (in particular autosomes) can be devoid of heterochromatin [13, 15, 37]. This distribution substantially differs from what observed in monocentric chromosomes, where the heterochromatic regions typically occupy specific zones of all chromosomes, corresponding to centromeres [38].

Lastly, no specific local DNA features that control kinetochore assembly have been identified in *C. elegans*, *T. urticae* and *A. pisum* [2, 25, 39]. At present considering that the chromosome compaction during mitosis involves condensin complexes, it has been suggested that holocentric chromosomes could be more rigid. This structural difference has been suggested in view of the absence of several proteins from the condensin complex I in the *C. elegans* genome [33]. Indeed, condensin I is dispensable for chromosome compaction and its interaction with chromatin at the end of prophase compaction may stabilize anaphase chromosomes, allowing them to better resist the spindle forces [25, 33]. Even if this hypothesis is intriguing, different hortologues coding for the condensin I complex subunits have been found in *T. urticae* [25] and *A. pisum* genomes [39] suggesting that the absence of these proteins is not a general feature in holocentric chromosomes. At the same time, the presence of a different role of the condensin I complex subunits in different taxa, together with the scattered presence of holocentrism, could reinforce the hypothesis that holocentric chromosome probably arose multiple times during evolution [2, 5, 16].

The uniform distribution of genes in holocentric chromosomes of metazoan is intriguing also in view of some consequences that could be related to holocentrism. According to what observed in *C. elegans* (the biological model where holocentric chromosomes have been mostly studied) meiotic recombination is much higher on the autosome termini than in the central region of chromosomes [2]. In view of the uniform distribution of genes, holocentric chromosome could consist of separate regions of high and low rates of recombination that could be related to slow/fast rate of evolution. Interestingly, the conserved set of eukaryotic genes shared by yeast and *C. elegans *are largely present in the central region of the chromosomes [33].

The lepidopteran genomes show a very high evolution rate (approximately 2 breakages per Mb per Mya) [40], making their chromosome evolution faster than that of nematodes, themselves evolving fourfold faster than *Drosophila* species [41], whose chromosomes rearrange two orders of magnitude faster than those of mammals and faster than plant chromosomes [42]. This very high rate is clearly unrelted to both the generation time and the effective population size [43], because these life history traits can be very similar between Lepidoptera and Diptera. Considering that nematodes and Lepidoptera share the presence of holocentric chromosomes, it has been suggested that the scattered organization of centromeric determinants may lead to a greater genomic plasticity as chromosome fragments resulting from double-strand breaks can be maintained and reintegrated elsewhere [40].

A further element of interest that emerges from the analysis of data regarding the evolution of holocentric chromosomes in Lepidoptera is related to the comparison of macrosynteny (describing large chromosomal regions) and microsynteny, dealing with clusters of neighbouring genes [40]. As a whole, it emerges the presence of small synteny blocks in a background of high macrosynteny in Lepidoptera in contrasts with *Drosophila*, in which paracentric inversions are common and result in a lack of synteny [40, 44, 45].

## HOLOCENTRIC CHROMOSOMES AND MEIOSIS: FACING THE COST OF HOLOCENTRISM

According to microscopic observations made in the first half of the last century, in many metazoa possessing holocentric chromosomes (such as aphids, lecanoid coccids and acariform mites), the meiotic processes is different in respect to the meiosis of taxa with monocentric chromosomes [3, 46]. In particular, in some species meiosis has been classified as “inverted” since the segregation of homologue chromosomes is postponed until the second meiotic division, so that the reduction to haploidy is achieved only at the second meiotic division [3, 5, 46, 47].

The cytological analysis of inverted meiosis has been often difficult and unconvincing since meiotic holocentric chromosomes were highly condensed. However, a definitive evidence for the presence of inverted meioses has been reported in coccids, together with interesting suggestions about the meiotic behaviour of holocentric chromosomes [47].

What is the reason for inverted meiosis in holocentric chromosomes? In monocentric species, homologous chromosomes are held paired by sister chromatid cohesion distal to crossover points so that the reductional segregation is achieved at anaphase I concomitantly with the loss of sister chromatid cohesion along chromatid arms [2, 48, 49]. Sister chromatid cohesion is instead maintained by centromeres until anaphase II in order to segregate sister chromatids during the equational second meiotic division [48, 49]. The absence of a localized centromere in holocentric chromosomes would render unfeasible the preservation of sister chromatid cohesion until anaphase and consequently, an ordered reductional first division would be unattainable [50].

However, as reported in literature, not all holokinetic species exhibit inverted meiosis. In nematodes, for example, meiotic divisions follow the traditional order [16, 51], as well as in three species of the heteropteran *Triatoma* [52]. How can they face the absence of an inadequate sister chromatid cohesion? A possible solution to this problem was described in *C. elegans*, where meiotic holocentric chromosomes have localized chromosome-microtubule attachments and behave as functionally monocentric [50]. In particular, it appears that in *C. elegans* meiotic chromosomes two potential regions can act as localized meiotic centromeres [50]. During meiosis I, one of these centromeres, chosen at random, becomes active and mediates the interactions between chromosomes and spindle, whereas the second potential meiotic centromere is active during meiosis II [50].

Differences between mitosis and meiosis have also been observed in holocentric chromosomes of the nematode *Ascaris*, where the chromosome kinetic activity during meiosis is restricted to one heterochromatic end, making meiotic chromosomes essentially monocentric [16]. A restriction of the kinetic activity to the chromosome ends during meiosis has been observed also in heteropteran insects, where no kinetochore structures have been observed, and chromosomes have been regarded as telokinetic [53]. Similarly to *C. elegans*, both the chromosome ends can act as localized centromere separately so that the chromosome end, which was inactive at the first meiotic division, become active during the second one [8, 54-57].

Several proteins resulted involved in the functioning of *C. elegans *centromere and kinetochore and most of them have been identified by comparative genomics through their homology to components identified in monocentric organisms, including HCP-1, HIM-10, ZW10, CENP-A (HCP-3) and CENP-C (HCP-4) [51]. Interestingly, several papers assessed that the RNA interference (RNAi) technique could be very effective for determining the involvement of some proteins in the *C. elegans *centromeric or kinetochore functions [51]. This approach is very intriguing since it allows to examine the consequences of depleting a specific gene product even in biological models where mutants are not available so that RNA interference, combined to comparative genomics, could have a pivotal role for assessing the role of proteins putatively involved in the functioning of centromeres and kinetochores in holocentric chromosomes [51]. 

## FUTURE PERSPECTIVES

As reported in several species, holocentrism allows the evolution of chromosome number, mainly from chromosome ﬁssion and fusion (rather than duplication and deletion) attesting the potential for chromosome evolution also at a relatively reduced temporal and geographic scales thus making the study of holocentrism intriguing for better evaluating its implications in ecological adaptation and speciation.

The availability of an increasing number of wholly sequenced genomes of organisms with holocentric chromosomes opened several interesting hypotheses about the evolution of this unusual chromosomal architecture. In view of the presence of several ongoing projects (including those of the tick *Ixodes scapularis* *and Varroa destructor*) it could be possible in the next future to face the evolution of holocentric chromosome from a wider point of view. In particular, a comparative analysis of the presence and function of the condensin I complex subunits could give interesting replies. At the same time it could be interesting to determine the global genomic organization of putative centromeric structures comparing holocentric species by combining comparative bioinformatic and functional approaches, also including RNA interference experiments that greatly increased the comprehension of holocentrism in the nematode *C. elegans* [2].
